# Microswimmer Propulsion by Two Steadily Rotating Helical Flagella

**DOI:** 10.3390/mi10010065

**Published:** 2019-01-18

**Authors:** Henry Shum

**Affiliations:** Department of Applied Mathematics, University of Waterloo, Waterloo, ON N2L 3G1, Canada; henry.shum@uwaterloo.ca; Tel.: +1-519-888-4567 (ext. 39157)

**Keywords:** bacterial locomotion, microswimmer, wall effect, multiple flagella, magnetotactic bacteria, boundary element method

## Abstract

Many theoretical studies of bacterial locomotion adopt a simple model for the organism consisting of a spheroidal cell body and a single corkscrew-shaped flagellum that rotates to propel the body forward. Motivated by experimental observations of a group of magnetotactic bacterial strains, we extended the model by considering two flagella attached to the cell body and rotating about their respective axes. Using numerical simulations, we analyzed the motion of such a microswimmer in bulk fluid and close to a solid surface. We show that positioning the two flagella far apart on the cell body reduces the rate of rotation of the body and increases the swimming speed. Near surfaces, we found that swimmers with two flagella can swim in relatively straight trajectories or circular orbits in either direction. It is also possible for the swimmer to escape from surfaces, unlike a model swimmer of similar shape but with only a single flagellum. Thus, we conclude that there are important implications of swimming with two flagella or flagellar bundles rather than one. These considerations are relevant not only for understanding differences in bacterial morphology but also for designing microrobotic swimmers.

## 1. Introduction

The locomotion of microscopic organisms, such as bacteria, has been a topic of fascination and mathematical analysis for well over sixty years [1]. Apart from seeking fundamental knowledge about how these evolutionarily simple organisms move and interact with their environment, there has recently been great interest in mimicking or using bacteria as microrobots with biomedical and environmental monitoring applications [2,3,4,5,6]. Of particular interest are magnetotactic bacteria, which can readily be steered by applying magnetic fields [7,8]. Organisms that are not naturally magnetotactic can also be controlled with magnetic fields after incorporation of magnetic particles; this has recently been demonstrated with the alga *Chlamydomonas reinhardtii* [9]. Commonly studied magnetotactic bacteria include the strains MO-1 and MC-1 (*Magnetococcus marinus*), which are similar in morphology and differ from the species most widely studied in other contexts, such as *Escherichia coli*, *Bacillus subtilis*, or *Vibrio alginolyticus*.

One of the striking morphological differences is that MO-1 develops two flagellar bundles that emerge from well defined locations on the cell body [10,11]. Each bundle consists of seven flagellar filaments and numerous fibrils enveloped in a sheath [12]. This complex structure propels the bacterium at speeds of up to 300 μ m/s, an order of magnitude faster than many other flagellated bacteria.

Notably, the cells of MO-1 have been reported to swim in the direction of the short axis, with the two flagellar bundles on either side of this axis [11]. In contrast, models of bacterial propulsion generally consider motion approximately aligned with the long axis of the cell, as this is typically the case for other types of bacteria. A common model [13,14,15,16] consists of a spherical or prolate spheroidal body and a helical flagellum extending from the posterior pole of the body. This model captures the essential physics and successfully accounts for the main typical features of bacterial locomotion. In this study, we focused on two related characteristics pertaining to bacteria swimming in the presence of a solid surface: the apparent attraction to the surface and the circular trajectories usually observed near the surface.

The presence of a no-slip wall affects the flow field around the bacterium and perturbs the swimming trajectory. The dominant far-field flow generated by a swimming bacterium is that of a force dipole, which experiences a hydrodynamic attraction to walls if the bacterium is moving parallel to the wall [17]. This explains the typical observation of accumulation of bacteria at surfaces. However, theoretical analysis shows that accumulation is not universal. Some bacterial shapes, particularly those with highly elongated cell bodies, are expected to result in escape from surfaces [15].

Separate from the attraction to or escape from surfaces is the curvature of swimming paths when the bacterium is (transiently) close to the wall. When a bacterium is parallel and close to a wall, the side of the bacterium closer to the wall experiences greater drag than the side further from the wall. The rotary motion of the flagella and counter-rotation of the cell body cause the body and flagella to “roll” sideways in opposite directions near a wall [18]. This leads to a constant turning motion, observed as a circular path. There is a strong correlation between the magnitude of curvature and the proximity to the surface [16]. The bacterial geometry affects the observed curvature in two ways: by directly determining the torque on the swimmer at a fixed distance from the wall, and by influencing the distance from the wall through attraction or escape.

Interestingly, experiments with MC-1 near flat surfaces do not indicate a tendency for such circular motion [19], suggesting that either the bacteria swim away from surfaces or that they exhibit small curvatures even close to a surface. Motivated by this unexpected behaviour at surfaces, we examined the simulated motion of bacteria with two flagella (representing the two flagellar bundles of MO-1 and MC-1) and compared the results to the well-studied model of propulsion by a single flagellum. We considered swimming in two environments: far from any obstructions (bulk fluid) and close to a solid wall. The former scenario has previously been modelled for bacteria with multiple flagella [20,21,22,23]. In bulk fluid, we found that the placement of the flagella significantly affects the swimming speed and the rate of rotation of the cell body as it swims. Near a wall, we show that swimming trajectories can change qualitatively depending on the positions and directions of the flagella. Intriguingly, the model bacteria can swim in orbits curving in either direction and with various curvatures. Moreover, we demonstrate that, for certain configurations of the flagella, the bacterium tends to swim away from walls.

These results are important because they emphasize the significance of detailed consideration of morphology. In particular, we illustrate an example in which the common modelling simplification of treating multiple flagella as a single rotating structure is inadequate.

## 2. Modelling and Methods

### 2.1. Geometric and Kinematic Model

**Figure 1:**
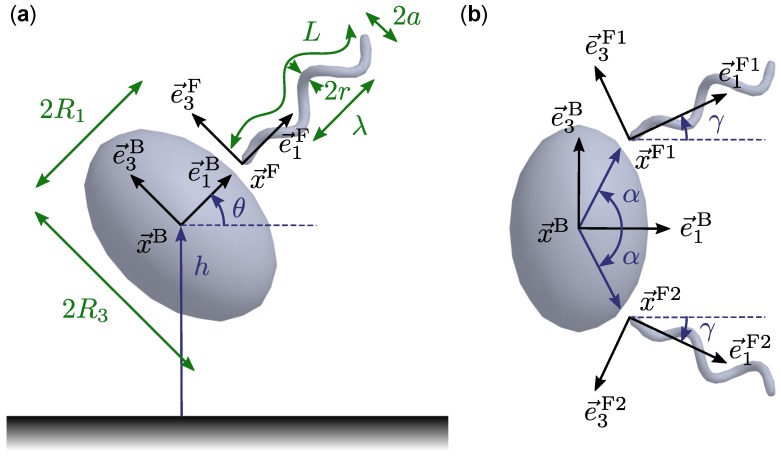
(**a**) Schematic of the one-tailed model bacterium near a wall with geometrical parameters labelled. The reference points and basis vectors (${\vec{e}}_{2}^{B}$ and ${\vec{e}}_{2}^{F}$ omitted for clarity) as well as the height *h* and inclination angle θ relative to the wall are also indicated. (**b**) Schematic of the two-tailed model bacterium showing the reference points and basis vectors of the body and two tails (omitting out-of-plane vectors for clarity) as well as the defining angles α and γ. Here, α=0.35π and γ=0.15π. The body and flagellum shape illustrated are those used in the presented results.

The bacterial model we considered consists of a prolate spheroidal cell body and two identical flagella, or tails, that are helical in shape except near the end that attaches to the cell body (the proximal end). The formulation is similar or equivalent to those used in previous theoretical studies of bacteria with one or multiple rigid flagella [14,15,16,20,21,22,23,24]. Following Higdon [13], the helical amplitude was modified so that the proximal end is parallel to and positioned on the central axis of the helix, as illustrated in Figure 1. The cell body and flagella are each rigid structures and so have fixed shapes in the (moving) reference frame of the respective structure. We chose the origin of the cell body to be its centre, denoted ${\vec{x}}^{B}$, and the three orthonormal basis vectors associated with the body are ${\vec{e}}_{j}^{B}$, j=1,2,3. An arbitrary point $\vec{x}={\vec{x}}^{B}+\xi_{1}{\vec{e}}_{1}^{B}+\xi_{2}{\vec{e}}_{2}^{B}+\xi_{3}{\vec{e}}_{3}^{B}$ on the surface of the cell body satisfies

(1) $${\left(\frac{\xi_{1}}{R_{1}}\right)}^{2}+{\left(\frac{\xi_{2}}{R_{2}}\right)}^{2}+{\left(\frac{\xi_{3}}{R_{3}}\right)}^{2}=1,$$

where we imposed $R_{1}=R_{2}$ for spheroids and non-dimensionalized all length scales in the system such that $R_{1}^{2}R_{3}=1$ in dimensionless units. The aspect ratio of the cell body is defined as $\zeta =R_{3}/R_{1}$ and is greater than unity for prolate spheroids.

Each flagellum is a long spherocylinder of radius *r* deformed so that its centreline follows a specified shape while transverse cross sections remain undeformed and perpendicular to the centreline. For the flagellum indexed by k=1,2, the reference point is ${\vec{x}}^{Fk}$ and the orthonormal basis vectors are ${\vec{e}}_{j}^{Fk}$, j=1,2,3. In the reference frame of the flagellum, the centreline is given by

(2) $$\begin{array}{ccc} \xi_{1}\left(s\right) & = & s, \end{array}$$

(3) $$\begin{array}{ccc} \xi_{2}\left(s\right) & = & a\left(1-e^{-k_{E}^{2}s^{2}}\right)\cos\left(2\pi s/\lambda \right), \end{array}$$

(4) $$\begin{array}{ccc} \xi_{3}\left(s\right) & = & a\left(1-e^{-k_{E}^{2}s^{2}}\right)\sin\left(2\pi s/\lambda \right), \end{array}$$

where $0\leq s\leq \xi_{\max}$, *a* is the maximal amplitude, $k_{E}$ is the amplitude growth factor, and λ is the helical pitch. We present results for cases with λ>0, which correspond to right handed helical filaments.

The positions ${\vec{x}}^{Fk}$ and axial directions ${\vec{e}}_{1}^{Fk}$ of the flagella are fixed relative to the cell body, as shown in Figure 1. The configuration of flagellum 1 is specified by two angles, α and γ. The direction of the flagellum axis is defined by γ, the angle from ${\vec{e}}_{1}^{B}$ to ${\vec{e}}_{1}^{F1}$. The angle α defines a point on the cell body surface and we chose ${\vec{x}}^{F1}$ to be offset from this point by a short distance $l_{h}$ in the outward normal direction. The purpose of this offset is to separate the flagellum from the cell body so that unphysical intersections are avoided. Note, however, that certain combinations of α and γ are not permissible because part of the flagellum would intersect the cell body or the other flagellum. We considered only symmetric flagellum configurations, where the second flagellum is a rotation of the first by the angle π around the ${\vec{e}}_{1}^{B}$ axis of the body.

In this study, we considered only one set of geometrical parameter values, listed in Table 1. The cell length and width and the diameter of the flagellar bundle were based on available measurements for MO-1 (1.85 μ m, 1.33 μ m, and 100 nm, respectively) [10,25]. Other parameters, such as the flagellar length and helical pitch during swimming, are not currently available in the literature for MO-1 or similar strains. Our parameters are consistent with values obtained by fitting experimental data [21] except that we considered a smaller helical amplitude so that we can vary α and γ over a wider range and investigate the influence of these two parameters. Given the uncertainty of physiological parameter values, we do not claim that our model accurately describes any particular strain of bacterium. Rather, we aimed to uncover some of the possible behaviours resulting from this mode of motility in biological or engineered swimmers.

The rigid body motion of the cell body is described by a translational velocity vector $\vec{U}$ and a rotational velocity vector $\vec{\Omega }$. The reference frame of the body moves relative to the stationary frame according to

(5) $$\begin{array}{ccc} \frac{d{\vec{x}}^{B}}{dt} & = & \vec{U}, \end{array}$$

(6) $$\begin{array}{ccc} \frac{d{\vec{e}}_{j}^{B}}{dt} & = & \vec{\Omega }\times {\vec{e}}_{j}^{B},\;j=1,2,3. \end{array}$$

An arbitrary point $\vec{x}$ on the surface of the body has the instantaneous velocity

(7) $$\vec{v}\left(\vec{x}\right)=\vec{U}+\vec{\Omega }\times \left(\vec{x}-{\vec{x}}^{B}\right).$$

In addition to following the rigid body motion of the cell body, the flagella rotate about their respective axes ${\vec{e}}_{1}^{Fk}$ with a fixed scalar rate ω=−2π in units of inverse dimensionless time, i.e.,

(8) $$\begin{array}{ccc} \frac{d{\vec{x}}^{Fk}}{dt} & = & {\vec{U}}^{Fk}=\vec{U}+\vec{\Omega }\times \left({\vec{x}}^{Fk}-{\vec{x}}^{B}\right), \end{array}$$

(9) $$\begin{array}{ccc} \frac{d{\vec{e}}_{j}^{Fk}}{dt} & = & {\vec{\Omega }}^{Fk}\times {\vec{e}}_{j}^{Fk}=\left(\vec{\Omega }+\omega {\vec{e}}_{1}^{Fk}\right)\times {\vec{e}}_{j}^{Fk},\;j=1,2,3. \end{array}$$

A point $\vec{x}$ on the surface of flagellum *k* has the instantaneous velocity

(10) $$\vec{v}\left(\vec{x}\right)=\vec{U}+\vec{\Omega }\times \left(\vec{x}-{\vec{x}}^{B}\right)+\omega {\vec{e}}_{1}^{Fk}\times \left(\vec{x}-{\vec{x}}^{Fk}\right).$$

### 2.2. Dynamics

Given a fixed motor frequency ω, we determined the unknowns $\vec{U}$ and $\vec{\Omega }$ by considering the fluid flow around the swimming model bacterium. We assumed that the flow field $\vec{u}$ is described by the incompressible Stokes equations, given in dimensionless form by

(11) $$-\nabla p+\nabla^{2}\vec{u}=\vec{0},\;\nabla \cdot \vec{u}=0,$$

where *p* is the pressure field. We imposed no-slip boundary conditions for the fluid on the surface of the bacterial cell body and flagella, i.e., the fluid velocities on these boundaries are given by Equations (7) and (10), respectively. We did not consider ambient flows, thus we required the flow velocity to decay far away from the swimmer. For some of our results, we simulated the motion of the swimmer near a flat, no-slip wall. In this case, we have the additional boundary condition $\vec{u}\left(x,y,z\right)=\vec{0}$ on the plane z=0.

The fluid exerts a distribution of viscous stress $\vec{f}$ over the surface *S* of the bacterium body and flagella. The net hydrodynamic force and torque are, respectively, given by ${\vec{F}}^{\mathrm{hyd}}=\oint_{S}\vec{f}\left(\vec{x}\right)\;dS\left(\vec{x}\right)$ and ${\vec{M}}^{\mathrm{hyd}}=\oint_{S}\left(\vec{x}-{\vec{x}}^{B}\right)\times \vec{f}\left(\vec{x}\right)\;dS\left(\vec{x}\right)$. Neglecting the inertia of the bacterium, we imposed the force and torque balance conditions

(12) $${\vec{F}}^{\mathrm{hyd}}+{\vec{F}}^{\mathrm{ext}}=\vec{0},\;{\vec{M}}^{\mathrm{hyd}}+{\vec{M}}^{\mathrm{ext}}=\vec{0},$$

where ${\vec{F}}^{\mathrm{ext}}$ and ${\vec{M}}^{\mathrm{ext}}$ are the net external force and torque acting on the swimmer, respectively. These external effects could include forces applied by an optical trap or a magnetic torque, if the bacterium has a magnetic dipole moment, for example. In the present work, we considered only contributions from short ranged repulsion when the bacterium is close to a wall. As in previous studies [26,27], we applied a normally directed repulsive force between the nearest points of the interacting surfaces (the cell body or a flagellum and the wall) of the form

(13) $${\vec{F}}^{\mathrm{rep}}=\alpha_{1}\frac{\alpha_{2}e^{-\alpha_{2}d}}{1-e^{-\alpha_{2}d}}{\vec{e}}_{z},$$

where *d* is the minimum separation between the wall and the swimmer structure. We set $\alpha_{1}=10$ and $\alpha_{2}=100$, which maintained minimum separations in the range 0.02–0.05 in typical simulations. With these force parameters, the repulsion is negligible when d>0.1 so the swimmer experiences only hydrodynamic forces when it is further than this distance from the wall.

### 2.3. Numerical Methods

We used a boundary element method (BEM) to solve the equations of Stokes flow subject to the given boundary conditions and constraints of force and torque balance. The BEM has been extensively applied to models of microorganism locomotion [14,15,26]. Given an instantaneous configuration (i.e., position and orientation) of the swimmer, the BEM determines $\vec{U}$ and $\vec{\Omega }$ as follows.

The solution for the flow field satisfying the governing equations from Section 2.2 can be expressed as the boundary integral equation [28],

(14) $$\vec{u}\left(\vec{x}\right)=-\frac{1}{8\pi }\oint_{S}\vec{f}\left(\vec{y}\right)\cdot G\left(\vec{y},\vec{x}\right)dS\left(\vec{y}\right),$$

where the tensor *G* is the Stokeslet Green’s function. In unbounded fluid, the components of *G* are defined by $G_{ij}\left(\vec{y},\vec{x}\right)=\frac{\delta_{ij}}{∥\vec{y}-\vec{x}∥}+\frac{\left(y_{i}-x_{i}\right)\left(y_{j}-x_{j}\right)}{{∥\vec{y}-\vec{x}∥}^{3}}$, whereas, in a half-space domain (with a no-slip boundary at z=0), the modified Green’s function given by Blake [29] was used.

For the numerical procedure, the surface of the swimmer (body and flagella) was discretized by a mesh of quadratic triangular elements. Equation (14) was imposed on the *N* mesh nodes of the swimmer, replacing the integral with its approximation using Gauss–Legendre quadrature over the surface mesh elements. The values of the traction vector field $\vec{f}$ at the *N* mesh nodes were treated as unknowns, as were the swimmer velocities $\vec{U}$ and Ω. In conjunction with the force and torque balance equation (Equation (12)), the system consists of 3N+6 linear equations, which can be solved to obtain the 3N+6 unknowns.

The linear and rotational velocities $\vec{U}$ and $\vec{\Omega }$ were then used to evolve the position and orientation of the model bacterium by a trapezoidal time-stepping algorithm [30].

## 3. Results

### 3.1. Swimming in Bulk Fluid

**Figure 2:**
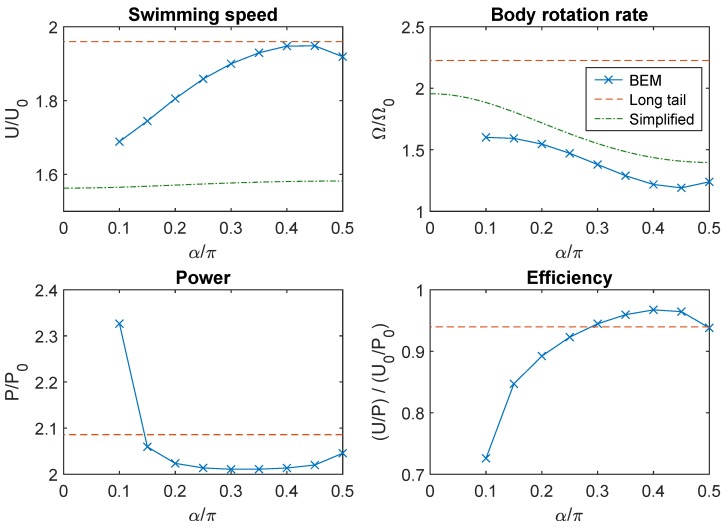
Swimming speed, body rotation rate, power and power-efficiency as functions of α for fixed γ=0. Solid curves marked with crosses are results of boundary element method (BEM) simulation. The dashed horizontal lines (labelled “Long tail”) indicate the values for a one-tailed swimmer (α=0 fixed) with flagellum length doubled. The swimming speeds and body rotation rates calculated by Equations (25) and (26), respectively, are also plotted (labelled “Simplified”).

In the absence of walls, the symmetry of the model swimmer led to translational motion in a straight line parallel to the ${\vec{e}}_{1}^{B}$ axis, i.e., $\vec{U}=U{\vec{e}}_{1}^{B}$ (see Video S1 for an example of swimming motion). The swimming speed *U* typically exhibited small amplitude periodic oscillations in time as the flagella rotated about their respective axes. The rotational velocity vector $\vec{\Omega }=\Omega {\vec{e}}_{1}^{B}$ was likewise aligned with the body axis and oscillated with a small amplitude. Using the set of parameter values listed in Table 1 for the shape of the body and flagella, we simulated the swimming motion of bacteria with two tails for 0≤α≤π/2 and γ=0. The average swimming speeds $\bar{U}$ and body rotation rates $\bar{\Omega }$ are presented as functions of α in Figure 2. These quantities were normalized by the respective values $U_{0}$ and $\Omega_{0}$ for a reference swimmer with a single flagellum of the same shape at α=γ=0. Note that, for swimmers with two flagella, values of α below a threshold were not permitted because the flagella would overlap.

As shown in Figure 2, the two-tailed swimmer was faster than the one-tailed swimmer for all tested values of α. The swimming speed increased with α and reached a peak at around α=0.45π before decreasing slightly. The highest swimming speed was $\bar{U}/U_{0}\approx 1.95$, meaning that the speed was almost doubled by doubling the number of flagella from one to two. For comparison, we also simulated a swimmer with a single flagellum of length double that of the flagella in the two-tailed model. This had a speed comparable to the maximum speed of the two-tailed swimmer.

The rotation rates of the body were also higher for two-tailed swimmers than for one-tailed swimmers. In contrast with the trend for swimming speeds, $\bar{\Omega }$ decreased with α and reached a minimum at around α=0.45π (Figure 2). The minimum rotation rate was $\bar{\Omega }/\Omega_{0}\approx 1.20$. For comparison, a swimmer with a single, long flagellum rotated with a rate more than double that of the reference one-tailed swimmer.

We defined the power dissipated (through rotation of the flagella under the action of torques applied by flagellar motors) by

(15) $$P=\sum_{k=1}^{N_{\mathrm{tail}}}\omega {\vec{e}}_{1}^{Fk}\cdot \oint_{F^{k}}\left(\vec{x}-{\vec{x}}^{Fk}\right)\times \vec{f}\left(\vec{x}\right)\;dS\left(\vec{x}\right),$$

where each integral is taken over the surface of flagellum *k* and $\vec{f}$ is the hydrodynamic traction field on the surface. The dependence of power and efficiency (U/P) on α are shown in Figure 2. For all tested values of α, the power required was greater than double that required for a single flagellum. There was relatively little variation with α except that the power dissipated increased rapidly as α approached the lower limit for valid tail configurations. In this limit, the two flagella were almost in contact, so there was more viscous resistance to relative motion between them. When the flagella were near the lateral poles (α≈0.5π), the proximal part of the flagellum was close to the surface of the cell body, resulting in a similar increase in power consumption. Swimming efficiency was low for small values of α and peaked at around α=0.4π with a value 97% of the efficiency for the swimmer with one flagellum.

### 3.2. Analytical Results from a Simplified Hydrodynamic Model

To understand the dependence of swimming speed and rotation rate on the angle α, we used a simple model for flagellar propulsion based on resistance coefficients of bodies in Stokes flow. Similar treatments have previously been considered for swimmers with one tail [18,31]. By linearity of the fluid flow equations, the hydrodynamic force and torque on an arbitrary rigid body are related to its translational and rotational velocity by [32]

(16) $$\left[\begin{array}{c} \vec{F}\\ \vec{M} \end{array}\right]=-\left[\begin{array}{cc} A & B\\ B^{T} & D \end{array}\right]\left[\begin{array}{c} \vec{U}\\ \vec{\Omega } \end{array}\right]$$

where A, B, and D are 3×3 resistance matrices that depend on the shape of the body and $B^{T}$ denotes the transpose of B. We applied this formulation to the cell body and two flagella as separate objects. The matrices for the spheroidal body could be constructed from known analytical results in Stokes flow [33] and the corresponding matrices for the flagella were computed using the BEM. An alternative approach could be to use resistive force theory to estimate the resistance coefficients for the flagella [18,34].

Some of the components of the resistance matrices were zero by symmetry or, for the flagella, averaged out to zero over a revolution about the tail axis [18]. Suppose that the translational and rotational velocity vectors for a swimming bacterium are parallel to ${\vec{e}}_{1}^{B}$. For simplicity, consider γ=0 so that the rotations of the flagella are also in the ${\vec{e}}_{1}^{B}$ direction. In the notation of Section 2, we have

(17) $$\vec{U}=U{\vec{e}}_{1}^{B},\;\vec{\Omega }=\Omega {\vec{e}}_{1}^{B},\;{\vec{U}}^{Fk}=U{\vec{e}}_{1}^{B}+\vec{\Omega }\times \left({\vec{x}}^{Fk}-{\vec{x}}^{B}\right),\;{\vec{\Omega }}^{Fk}=\left(\Omega +\omega \right){\vec{e}}_{1}^{B}$$

The components of force and torque along the ${\vec{e}}_{1}^{B}$ axis are given by

(18) $$F_{1}^{B}=-a^{B}U,\;M_{1}^{B}=-d^{B}\Omega ,$$

for the cell body and

(19) $$F^{F}k_{1}=-a^{F}U-b^{F}\left(\Omega +\omega \right),\;M^{F}k_{1}=-b^{F}U-d^{F}1\left(\Omega +\omega \right),$$

for the flagella. When the flagella are not on the central axis of the swimmer, rotation of the cell body also leads to linear motion of the flagella in the ${\vec{e}}_{2}^{B}$ direction. Writing $\left({\vec{x}}^{F1}-{\vec{x}}^{B}\right)=l_{∥}{\vec{e}}_{1}^{B}+l_{⊥}{\vec{e}}_{3}^{B}$ and $\left({\vec{x}}^{F2}-{\vec{x}}^{B}\right)=l_{∥}{\vec{e}}_{1}^{B}-l_{⊥}{\vec{e}}_{3}^{B}$, the force components in this direction satisfy

(20) $$F_{2}^{F1}=a^{F}{}_{⊥}\Omega l_{⊥}=-F_{2}^{F2}.$$

The equations for force and torque balance of the swimmer (expressed in the reference frame of the cell body), respectively, read

(21) $$\begin{array}{ccc} {\vec{F}}^{B}+\sum_{k=1}^{N_{\mathrm{tail}}}{\vec{F}}^{Fk} & = & \vec{0}, \end{array}$$

(22) $$\begin{array}{ccc} {\vec{M}}^{B}+\sum_{k=1}^{N_{\mathrm{tail}}}\left({\vec{M}}^{Fk}+\left({\vec{x}}^{Fk}-{\vec{x}}^{B}\right)\times {\vec{F}}^{Fk}\right) & = & \vec{0}. \end{array}$$

For a swimmer with one tail and α=0 (i.e., $l_{⊥}=0$), we obtained the expressions for the body rotation rate and swimming speed [31]

(23) $$\begin{array}{ccc} \Omega_{0} & = & -\frac{\hat{a}d^{F}-{\left(b^{F}\right)}^{2}}{\hat{a}\hat{d}-{\left(b^{F}\right)}^{2}}\omega \approx -\frac{d^{F}}{\hat{d}}\omega , \end{array}$$

(24) $$\begin{array}{ccc} U_{0} & = & -\frac{b^{F}}{\hat{a}}\left(\Omega_{0}+\omega \right)=-\frac{b^{F}d^{B}}{\hat{a}\hat{d}-{\left(b^{F}\right)}^{2}}\omega \approx -\frac{b^{F}d^{B}}{\hat{a}\hat{d}}\omega , \end{array}$$

where $\hat{a}=a^{B}+a^{F}$, $\hat{d}=d^{B}+d^{F}$, and the approximations were obtained by considering ${\left(b^{F}\right)}^{2}\ll a^{F}d^{F}$, as anticipated by Purcell [31]. Since Purcell did not provide any justification for this approximation, we verified that it was reasonable for our flagellum shape by determining that ${\left(b^{F}\right)}^{2}/\left(a^{F}d^{F}\right)=0.009$. Similarly, the rotation rate and speed for a swimmer with two flagella are

(25) $$\begin{array}{ccc} \Omega & = & -\frac{\tilde{a}{\tilde{d}}^{F}-{\left({\tilde{b}}^{F}\right)}^{2}}{\tilde{a}\tilde{d}-{\left({\tilde{b}}^{F}\right)}^{2}+\tilde{a}{\tilde{a}}_{⊥}^{F}l_{⊥}^{2}}\omega \approx -\frac{{\tilde{d}}^{F}}{\tilde{d}+{\tilde{a}}_{⊥}^{F}l_{⊥}^{2}}\omega ,\; \end{array}$$

(26) $$\begin{array}{ccc} U & = & -\frac{{\tilde{b}}^{F}}{\tilde{a}}\left(\Omega +\omega \right)=-\frac{{\tilde{b}}^{F}\left(d^{B}+{\tilde{a}}_{⊥}^{F}l_{⊥}^{2}\right)}{\tilde{a}\tilde{d}-{\left({\tilde{b}}^{F}\right)}^{2}+\tilde{a}{\tilde{a}}_{⊥}^{F}l_{⊥}^{2}}\omega \approx -\frac{{\tilde{b}}^{F}\left(d^{B}+{\tilde{a}}_{⊥}^{F}l_{⊥}^{2}\right)}{\tilde{a}\left(\tilde{d}+{\tilde{a}}_{⊥}^{F}l_{⊥}^{2}\right)}\omega , \end{array}$$

where ${\tilde{a}}_{⊥}^{F}=2a_{⊥}^{F}$, $\tilde{a}=a^{B}+2a^{F}$, ${\tilde{b}}^{F}=2b^{F}$, ${\tilde{d}}^{F}=2d^{F}$, and $\tilde{d}=d^{B}+2d^{F}$.

For the geometrical parameters in Table 1, we found that ${\tilde{b}}^{F}<0$, while all other coefficients in Equations (25) and (26) were positive. Hence, we could deduce that Ω is opposite in sign to ω (the body and flagella counter-rotate) and that the magnitude of the body rotation rate decreases with $l_{⊥}$ and is always less |ω|. We also note that *U* is negative (forward swimming in our formulation) if ω<0 and that |U| increases as $l_{⊥}$ increases, saturating at the value $|U^{\max}\left|=\right|{\tilde{b}}^{F}\omega |/\tilde{a}$ as $l_{⊥}\to \infty$.

If the flagella were attached to the surface of the cell body, then the transverse displacement $l_{⊥}$ would be related to the angle α and the dimensions $R_{1}$ and $R_{3}$ by the formula $l_{⊥}=R_{1}\sin\alpha {\left(1-e^{2}\sin^{2}\alpha \right)}^{-1/2}$, where $e=\sqrt{1-R_{1}^{2}/R_{3}^{2}}$ is the eccentricity of the spheroidal cell body. This allowed us to explicitly express the dependence of *U* and Ω on α. Figure 2 compares $U/U_{0}$ and $\Omega /\Omega_{0}$ estimated by this approach with the results from our BEM simulations. There was good agreement for the body rotation rates but the simplified theory under-predicted the enhancement of swimming speed from having two tails.

### 3.3. Swimming Near a No-Slip Wall

We next examined the behaviour of the model bacteria in the presence of a no-slip ($\vec{u}=\vec{0}$) wall at z=0. The cell body and flagella shapes were the same as in the bulk fluid case but we varied both α and γ to determine how the configuration of the two flagella affects propulsion near the wall. In each case, the swimmer started at height $h={\vec{x}}^{B}\cdot {\vec{e}}_{z}=3$ and inclination angle $\theta =\arcsin({\vec{e}}_{1}^{B}\cdot {\vec{e}}_{z})=\pi /4$ so the swimming direction was angled towards the wall. In general, it is possible that the long-time behaviour depends on the initial height and inclination angle. For example, a previous simulation study [35] shows that some swimmers escape from the wall if they start with a shallow angle of inclination but become hydrodynamically bound to the wall if they approach with a steeper angle. However, this type of behaviour was only found in a narrow range of parameter combinations between swimmers that always escape and those that are always hydrodynamically bound to the wall. In the current study, we started with θ=π/4, which we expected to be sufficiently steep to reach the bound state, if one exists.

**Figure 3:**
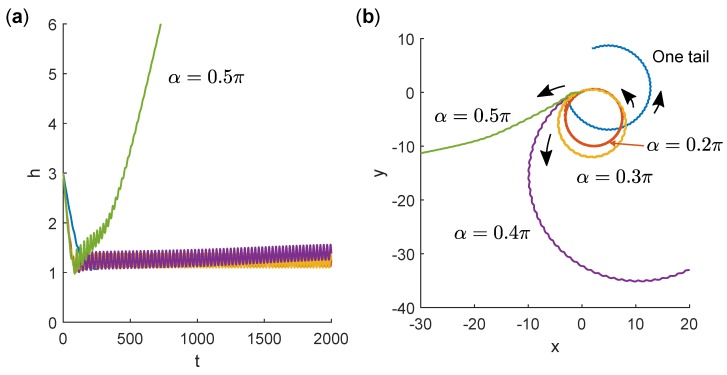
(**a**) Time series of height *h* of swimmers with different α values near a wall. Trajectories all begin with h=3 and θ=π/4. In all cases, γ=0. (**b**) Projections of trajectories in the plane of the wall. Trajectories all begin at x=y=0 and colours correspond to the same simulations as in (**a**).

Trajectories were computed up to dimensionless time $t_{\mathrm{final}}=2000$, which corresponds to 2000 revolutions of the flagella. For comparison, we also included results for a swimmer with one flagellum at α=γ=0. This swimmer became bound to the wall and swam in a counter-clockwise circular orbit close to the wall, as shown in Figure 3.

Fixing γ=0 and varying α for a two-tailed swimmer, we found that small values of α led to similar bound, circular motion. The average height of the bacterium above the wall was almost invariant but the radius of curvature of the orbit increased with α until α≥0.4π, for which we found that the swimmer escaped from the wall. In the escaping trajectories, interestingly, the *x*–*y* projection of the path curved in the counter-clockwise direction as the bacterium approached the wall and in the clockwise direction as the bacterium escaped the wall. Eventually, the path became straight as the effect of the wall diminished with separation.

**Figure 4:**
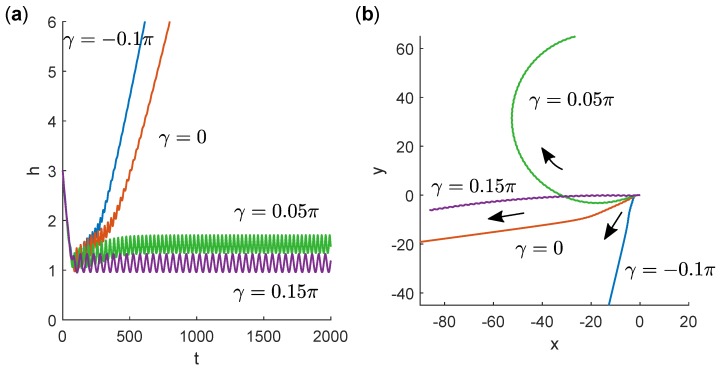
(**a**) Time series of height *h* of swimmers with different γ values near a wall. Trajectories all begin with h=3 and θ=π/4. In all cases, α=0.475π. (**b**) Projections of trajectories in the plane of the wall. Trajectories all begin at x=y=0 and colours correspond to the same simulations as in (**a**).

In Figure 4, we present trajectories for fixed α=0.475π and varying γ. The swimmer escaped from the wall for γ≤0 (tails parallel or pointing inward) and was bound in circular orbits at the wall for γ≥0.05π (tails pointing outward). Among the bound, circular orbits, curvature in both directions was possible. For γ=0.05π, the swimmer orbited in the clockwise sense while, for γ=0.15π, the path curved in the counter-clockwise direction (with a large radius of curvature).

**Figure 5:**
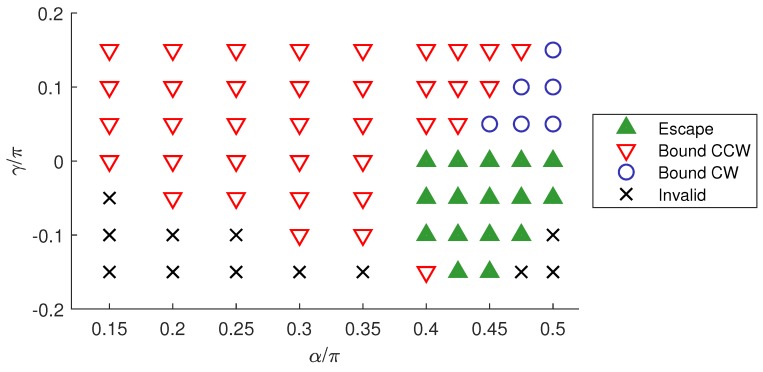
Phase map of two-tailed swimmer behaviour near a wall. Swimmers can escape from the wall, swim in stable counter-clockwise orbits near the wall (“Bound CCW”) or swim in stable clockwise orbits near the wall (“Bound CW”). Some configurations are invalid due to intersections between flagella or between the flagellum and cell body.

The behaviour of two-tailed bacteria at a wall for different combinations of α and γ is summarized in Figure 5. The predominant behaviour was counter-clockwise orbiting along the surface as in the one-tailed case. When γ≲0 and α was large, such that the flagella were close to the lateral poles of the cell body, the swimmer tended to escape from surfaces. When α was large but γ was slightly above the threshold for wall escape, the swimmer became bound to the wall and swam in clockwise circles. Videos S2–S4, respectively, exemplify these three classes of behaviour.

## 4. Discussion

The aim of this study was to highlight some of the consequences of a particular mode of bacterial swimming using two backward-facing flagella that do not bundle together. The results presented can be interpreted both to understand the implications for bacteria with such a morphology and to aid the design of microrobotic swimmers with specific characteristic behaviours. One of the key findings is that placing the flagella far apart, so as to maximize $l_{⊥}$, significantly reduces the body rotation rate. A theoretical study [36] suggested that body rotation could be sufficient to cause bundling of flagella. Thus, reducing body rotation could be an effective means of preventing the two lateral flagella from bundling together.

Moreover, since this body rotation diminishes the net rotation of the flagella with respect to the ambient fluid, propulsive thrust is increased when body rotation is suppressed. The result is higher swimming speeds with two lateral flagella. Swimmers with a single flagellum or flagellar bundle could achieve similar speed ups by doubling the flagellar length or motor frequency. These strategies, however, entail much larger stresses on the flagellum, which might then require a stiffer structure to avoid instabilities [30,37,38]. Compared with these modified one-tail scenarios, the two-tailed swimmer has a much lower body rotation rate for the same swimming speed.

We remark that the simplified model, which does not account for hydrodynamic interactions among the components of the swimmer, yields a lower speed enhancement with two tails than the full BEM model. This suggests that the presence of one flagellum increases the thrust produced by the other, reminiscent of previously reported speed increases for a model bacterium swimming near a wall [24].

The reduced body rotation rate could confer another benefit, at least in an artificial setting. The two-tailed morphology in this study was motivated by a group of magnetotactic bacteria such as the strain MO-1. The cell’s magnetosome chain, which is responsible for its magnetic dipole moment, is generally at an oblique angle to the cell axis [7,21]. Hence, the orientation of the magnetic dipole moment precesses as the cell body rotates and, consequently, the magnetic torques experienced by bacteria are affected by the rate of body rotation due to flagellar propulsion. The reduced body rotation rate (for a given swimming speed) could allow better alignment of the magnetosomes with a magnetic field. In an application where magnetotactic bacteria are controlled by time-varying magnetic fields [8,21,39], this could result in greater manoeuvrability and higher step-out frequencies.

In the presence of walls, our results indicate that it is possible to achieve qualitatively distinct behaviour with two flagella. Depending on the positions and orientations of the two flagella with respect to the body, the swimmer can either remain trapped in curved orbits at the wall or escape back into the bulk fluid. Since many potential uses of microrobots would involve proximity to surfaces, it is important to understand how subtle changes in the design could alter the swimming motion. It may even be possible to exploit the dependence on configuration to dynamically control the motion of a two-tailed microrobot near a wall. A simple way to achieve this is through passive elastic deformations. The fluid flow generated by two parallel flagella tends to pull them together, contributing to flagellar bundling in peritrichous bacteria. If the filaments are sufficiently stiff and the flagella far apart, bundling will not occur but the tail axes will bend inward, i.e., γ would become more negative. Changes in the motor frequency would affect the resulting bend angle, thereby allowing the path curvature to be controlled or escape from a surface to be initiated. A similar effect of hook flexibility on near-wall behaviour was previously also described for swimmers with a single flagellum [30].

Bacteria with morphologies similar to our two-tailed model have been studied in many experimental works [10,11,19,21]. We are unaware of data or reports of such bacteria swimming in circles due to interactions with walls. In fact, even when the bacteria were observed between parallel surfaces, non-magnetic *M. marinus* did not exhibit noticeable circular orbits [19]. This behaviour contrasts with expectations based on a model bacterium with a single flagellum, suggesting that the two-tailed morphology may be an important factor in explaining the relatively straight trajectories of *M. marinus* near walls. Further studies are needed to verify whether the positioning of the flagella indeed influences the path curvature and attraction to walls, as indicated by our simulations.

While we are unaware of any measurements of flagellum orientation in swimming MO-1 or MC-1, it is unlikely that the two bundles point away from each other (γ>0) due to the tendency for attraction discussed above. Based on the presented results, this suggests that circles of the unconventional direction (clockwise in our model, counter-clockwise if the flagella are left-handed helices) would not be observed. We remark, however, that we have only considered one set of parameters for the body and tail shapes. A more thorough investigation of the switch in curvature and its dependence on geometrical parameters is still needed to evaluate whether this phenomenon could apply to known bacterial strains.

## Figures and Tables

**Table 1:** Parameters defining the shape of the bacterial cell body and flagella. The length scale for non-dimensionalization is $l^{*}=0.74\;\mu$ m.

Description	Symbol	Value (Dimensionless)	Value (Dimensional)
Body short semi-axis	$R_{1}$	0.874	0.65 μ m
Body aspect ratio	$\zeta =R_{3}/R_{1}$	1.50	1.50
Flagellum length	*L*	3.00	2.2 μ m
Filament radius	*r*	0.067	50 nm
Helical amplitude	*a*	0.200	0.15 μ m
Helical pitch	λ	1.00	0.74 μ m
Amplitude growth factor	$k_{E}$	0.333·2π/λ=2.09	2.8 μ m${}^{-1}$
Body–flagellum gap	$l_{h}$	2r=0.134	100 nm

## Supplementary Materials

The following are available online at http://www.mdpi.com/2072-666X/10/1/65/s1. Video S1: Swimming in bulk fluid (α=0.45π, γ=−0.1π), Video S2: Bound counter-clockwise at wall (α=0.3π, γ=0), Video S3: Escape from wall (α=0.5π, γ=0), Video S4: Bound clockwise at wall (α=0.475π, γ=0.05π).

## References

[1] Taylor G. (1951). Analysis of the Swimming of Microscopic Organisms. Proc. R. Soc. Lond. Ser. A.

[2] Peyer K.E., Zhang L., Nelson B.J. (2013). Bio-inspired magnetic swimming microrobots for biomedical applications. Nanoscale.

[3] Martel S. (2016). Swimming microorganisms acting as nanorobots versus artificial nanorobotic agents: A perspective view from an historical retrospective on the future of medical nanorobotics in the largest known three-dimensional biomicrofluidic networks. Biomicrofluidics.

[4] Singh A.V., Hosseinidoust Z., Park B.W., Yasa O., Sitti M. (2017). Microemulsion-Based Soft Bacteria-Driven Microswimmers for Active Cargo Delivery. ACS Nano.

[5] Klumpp S., Lefèvre C.T., Bennet M., Faivre D. (2018). Swimming with magnets: From biological organisms to synthetic devices. Phys. Rep..

[6] Alapan Y., Yasa O., Yigit B., Yasa I.C., Erkoc P., Sitti M. (2019). Microrobotics and Microorganisms: Biohybrid Autonomous Cellular Robots. Annu. Rev. Control Robot. Auton. Syst..

[7] Pan Y., Lin W., Li J., Wu W., Tian L., Deng C., Liu Q., Zhu R., Winklhofer M., Petersen N. (2009). Reduced Efficiency of Magnetotaxis in Magnetotactic Coccoid Bacteria in Higher than Geomagnetic Fields. Biophys. J..

[8] Yazdi S.R., Nosrati R., Stevens C.A., Vogel D., Davies P.L., Escobedo C. (2018). Magnetotaxis Enables Magnetotactic Bacteria to Navigate in Flow. Small.

[9] Santomauro G., Singh A.V., Park B.W., Mohammadrahimi M., Erkoc P., Goering E., Schütz G., Sitti M., Bill J. (2018). Incorporation of Terbium into a Microalga Leads to Magnetotactic Swimmers. Adv. Biosys..

[10] Lefèvre C.T., Bernadac A., Yu-Zhang K., Pradel N., Wu L.F. (2009). Isolation and characterization of a magnetotactic bacterial culture from the Mediterranean Sea. Environ. Microbiol..

[11] Zhang S.D., Petersen N., Zhang W.J., Cargou S., Ruan J., Murat D., Santini C.L., Song T., Kato T., Notareschi P., Li Y., Namba K., Gué A.M., Wu L.F. (2014). Swimming behaviour and magnetotaxis function of the marine bacterium strain MO-1: Magnetotaxis of MO-1. Environ. Microbiol. Rep..

[12] Ruan J., Kato T., Santini C.L., Miyata T., Kawamoto A., Zhang W.J., Bernadac A., Wu L.F., Namba K. (2012). Architecture of a flagellar apparatus in the fast-swimming magnetotactic bacterium MO-1. Proc. Natl. Acad. Sci. USA.

[13] Higdon J.J.L. (1979). The hydrodynamics of flagellar propulsion: helical waves. J. Fluid Mech..

[14] Phan-Thien N., Tran-Cong T., Ramia M. (1987). A boundary-element analysis of flagellar propulsion. J. Fluid Mech..

[15] Shum H., Gaffney E.A., Smith D.J. (2010). Modelling bacterial behaviour close to a no-slip plane boundary: The influence of bacterial geometry. Proc. R. Soc. A.

[16] Giacché D., Ishikawa T., Yamaguchi T. (2010). Hydrodynamic entrapment of bacteria swimming near a solid surface. Phys. Rev. E.

[17] Berke A.P., Turner L., Berg H.C., Lauga E. (2008). Hydrodynamic Attraction of Swimming Microorganisms by Surfaces. Phys. Rev. Lett..

[18] Lauga E., DiLuzio W.R., Whitesides G.M., Stone H.A. (2006). Swimming in Circles: Motion of Bacteria near Solid Boundaries. Biophys. J..

[19] Lin H.Y. (2017). Motility and Intelligence of Microorganisms in Microconfined Networks. Master’s Thesis.

[20] Hyon Y., Powers T.R., Stocker R., Fu H.C. (2012). The wiggling trajectories of bacteria. J. Fluid Mech..

[21] Yang C., Chen C., Ma Q., Wu L., Song T. (2012). Dynamic Model and Motion Mechanism of Magnetotactic Bacteria with Two Lateral Flagellar Bundles. J. Bionic Eng..

[22] Kanehl P., Ishikawa T. (2014). Fluid mechanics of swimming bacteria with multiple flagella. Phys. Rev. E.

[23] Riley E.E., Das D., Lauga E. (2018). Swimming of peritrichous bacteria is enabled by an elastohydrodynamic instability. arXiv.

[24] Ramia M., Tullock D., Phan-Thien N. (1993). The role of hydrodynamic interaction in the locomotion of microorganisms. Biophys. J..

[25] Lefèvre C.T., Santini C.L., Bernadac A., Zhang W.J., Li Y., Wu L.F. (2010). Calcium ion-mediated assembly and function of glycosylated flagellar sheath of marine magnetotactic bacterium. Mol. Microbiol..

[26] Ishikawa T., Hota M. (2006). Interaction of two swimming Paramecia. J. Exp. Biol..

[27] Shum H., Yeomans J.M. (2017). Entrainment and scattering in microswimmer-colloid interactions. Phys. Rev. Fluids.

[28] Kim S., Karrila S.J. (2005). Microhydrodynamics: Principles and Selected Applications.

[29] Blake J.R. (1971). A note on the image system for a stokeslet in a no-slip boundary. Math. Proc. Camb. Philos. Soc..

[30] Shum H., Gaffney E.A. (2012). The effects of flagellar hook compliance on motility of monotrichous bacteria: A modeling study. Phys. Fluids.

[31] Purcell E.M. (1997). The efficiency of propulsion by a rotating flagellum. Proc. Natl. Acad. Sci. USA.

[32] Happel J., Brenner H. (1965). Low Reynolds Number Hydrodynamics: With Special Applications to Particulate Media.

[33] Chwang A.T., Wu T.Y.T. (1975). Hydromechanics of Low-Reynolds-Number Flow. Part 2. Singularity Method for Stokes Flows. J. Fluid Mech..

[34] Gray J., Hancock G.J. (1955). The Propulsion of Sea-Urchin Spermatozoa. J. Exp. Biol..

[35] Shum H., Gaffney E.A. (2015). Hydrodynamic analysis of flagellated bacteria swimming near one and between two no-slip plane boundaries. Phys. Rev. E.

[36] Powers T.R. (2002). Role of body rotation in bacterial flagellar bundling. Phys. Rev. E.

[37] Vogel R., Stark H. (2012). Motor-driven bacterial flagella and buckling instabilities. Eur. Phys. J. E.

[38] Jawed M., Khouri N., Da F., Grinspun E., Reis P. (2015). Propulsion and Instability of a Flexible Helical Rod Rotating in a Viscous Fluid. Phys. Rev. Lett..

[39] Steinberger B., Petersen N., Petermann H., Weiss D.G. (1994). Movement of magnetic bacteria in time-varying magnetic fields. J. Fluid Mech..

